# Prognostic value of pretreatment platelet counts in lung cancer: a systematic review and meta-analysis

**DOI:** 10.1186/s12890-020-1139-5

**Published:** 2020-04-20

**Authors:** Yuan Yuan, Hai Zhong, Liang Ye, Qian Li, Surong Fang, Wei Gu, Yingying Qian

**Affiliations:** 1 Department of Respiratory and Critical Care Medicine, Nanjing First Hospital, Nanjing Medical University, 68 Changle road, Nanjing, 210006 Jiangsu China; 2 Department of Pulmonary and Critical Care Medicine, The Affiliated Jiangning Hospital of Nanjing Medical University, Nanjing, 210006 Jiangsu China

**Keywords:** Lung cancer, Platelet count, Prognosis, Meta-analysis

## Abstract

**Background:**

The prognostic value of elevated pretreatment platelet counts remains controversial in lung cancer patients. We performed the present meta-analysis to determine its precise role in these patients.

**Methods:**

We employed a multiple search strategy in the PubMed, EMBASE and Cochrane Library databases to identify eligible studies. Disease-free survival (DFS)/progression-free survival (PFS)/time to progression (TTP) and overall survival (OS) were used as outcomes with hazard ratios (HRs) and 95% confidence intervals (CIs). Heterogeneity among the studies and publication bias were also evaluated.

**Results:**

A total of 40 studies including 16,696 lung cancer patients were eligible for the analysis. Overall, the pooled analysis showed that compared with normal platelet counts, elevated pretreatment platelet counts were associated with poorer OS (HR = 1.54, 95% CI: 1.37–1.72, *P* < 0.001) and poorer DFS/PFS/TTP (HR = 1.62, 95% CI: 1.33–1.98, *P* < 0.001) in patients with lung cancer. In subgroup analyses, elevated pretreatment platelet counts were also associated with poorer OS and DFS/PFS/TTP in most subgroups. There was no evidence of publication bias.

**Conclusions:**

This meta-analysis revealed that elevated pretreatment platelet counts were an independent predictor of OS and DFS/PFS/TTP in lung cancer patients. Large-scale prospective studies and a validation study are warranted.

## Background

According to the Global Cancer Statistics 2018, lung cancer is the most common cancer (11.6% of total cancer cases) and the leading cause of cancer deaths (18.4% of the total cancer deaths) worldwide [1]. Non-small-cell lung cancer (NSCLC), the leading type of lung cancer, accounts for 80% of all cases. Although various therapies, such as surgery, radiotherapy, chemotherapy, targeted therapy, and the rising immunization therapy have emerged, they exhibit limited effects on lung cancer, and the prognosis of patients remains unsatisfactory, with five-year survival rates of 6.3% for small cell lung cancer (SCLC) and 18.2% for NSCLC [2–4]. Compared to treating advanced cancer, prevention is much better. Therefore, it is important to investigate novel prognostic factors to improve treatment therapies.

In the 1960s, Richard B. et al. suggested that platelets were correlated with cancer [5]. Tumour cells can secrete platelet agonists to induce platelet aggregation, which results in thrombocytosis by producing thrombopoietic cytokines such as interleukin (IL)-1, IL-3, IL-11, and particularly tumour-derived IL-6 [6–9]. Many studies have shown that thrombocytosis plays a role in cancer genesis and development [10, 11]. Increasing evidence has indicated that platelet count correlates with prognosis in various malignancies, such as lung, renal, gastric, colorectal and hepatocellular cancer and is considered a hallmark of cancer [12–16]. Additionally, the platelet count is convenient to perform, less expensive, and easily available. However, the current opinions about the correlation between platelet count and lung cancer prognosis are controversial. Some studies have identified that platelet count is a poor prognostic factor in NSCLC, while some suggest that platelet count has no association with lung cancer [11, 17–19]. Therefore, we conducted this meta-analysis to further investigate the prognostic value of pretreatment platelet counts for survival in lung cancer patients.

## Methods

### Search strategy

This meta-analysis was conducted and reported in accordance with the Preferred Reporting Items for Systematic Reviews and Meta-Analyses (PRISMA) guidelines (Additional file 1). A comprehensive search was conducted by searching databases including the PubMed, EMBASE and Cochrane Library databases using the following terms: (“thrombocytosis” or “thrombocytosis” or “thrombocythemia” or “platelet count” or “blood platelets” or “platelets”) and (“lung carcinoma” or “lung cancer” or “lung tumor” or “lung neoplasm”) and (“prognosis” or “prognostic” or “survival” or “outcome”) up to December 31, 2017.

### Selection criteria

The inclusion criteria for this meta-analysis were as follows: (1) the diagnosis of lung cancer was confirmed pathologically; (2) platelet count was measured before treatment; (3) hazard ratios (HRs) and their 95% confidence intervals (CIs) for platelet count were reported; (4) the cut-off value of platelet count was reported; and (5) the relationship between overall survival (OS) or disease-free survival (DFS)/progression-free survival (PFS)/time to progression (TTP) and platelet count was evaluated.

### Exclusion criteria

Articles were excluded if they met the following criteria: (1) articles were reviews, case reports, letters, editorials, or conference abstracts; (2) articles that were not written in English; (3) articles missing key information for evaluating the HR and its 95% CI; (4) studies based on cancer cells or animal models and irrelevant studies; and (5) studies by the same authors with similar or overlapping data. Two reviewers assessed the candidate articles independently. Any disputes were settled through discussion.

### Data extraction and quality assessment

Two reviewers independently extracted data from the selected literature and completed quality assessments. The first author name, year of publication, country of origin, number of enrolled patients, tumour type, clinical stage, cut-off value of platelet count, and outcomes were included as publication characteristics. HRs for OS and PFS and their 95% CIs were collected as result data. If the study provided both univariate analysis and multivariate analysis results, we took the results of multivariate analysis because multivariate analyses exclude correlated confounding factors and are more accurate. In addition, only one study (Holgersson G, 2012) categorized platelet count into three groups according to cut-off values (platelet count < 150, 150 < platelet count < 350, and platelet count > 350), and there were two HRs for OS. We extracted the HR that compared the group with 150 < platelet count < 350 and the group with platelet count > 350. We used the Newcastle-Ottawa Scale (NOS) scoring system to assess the quality of the included articles [20]. Two reviewers independently evaluated the quality of each included study. The judgement criteria include three aspects of evaluation: selection, comparability, and outcome between the case group and control group. Studies with higher scores had higher quality.

### Statistical analysis

The meta-analysis was conducted by STATA 12.0 software (Stata Corp, College Station, TX, USA). HRs and corresponding 95% CIs were used to analyse the association between platelet count and lung cancer. Cochrane’s Q test and the I2 statistic were used to evaluate the heterogeneity among the included studies [21]. I2 > 50% or *P*-value< 0.05 indicated heterogeneity in the studies [22, 23], and a random-effects model was adopted; otherwise, a fixed-effects model was used. Moreover, subgroup analysis was conducted to detect the potential source of heterogeneity. A *P*-value less than 0.05 indicated statistical significance. Publication bias was evaluated by Begg’s test and Egger’s regression test [24]. Additionally, sensitivity analysis was performed to check the stability of the results [25].

## Results

### Study characteristics

**Fig. 1:**
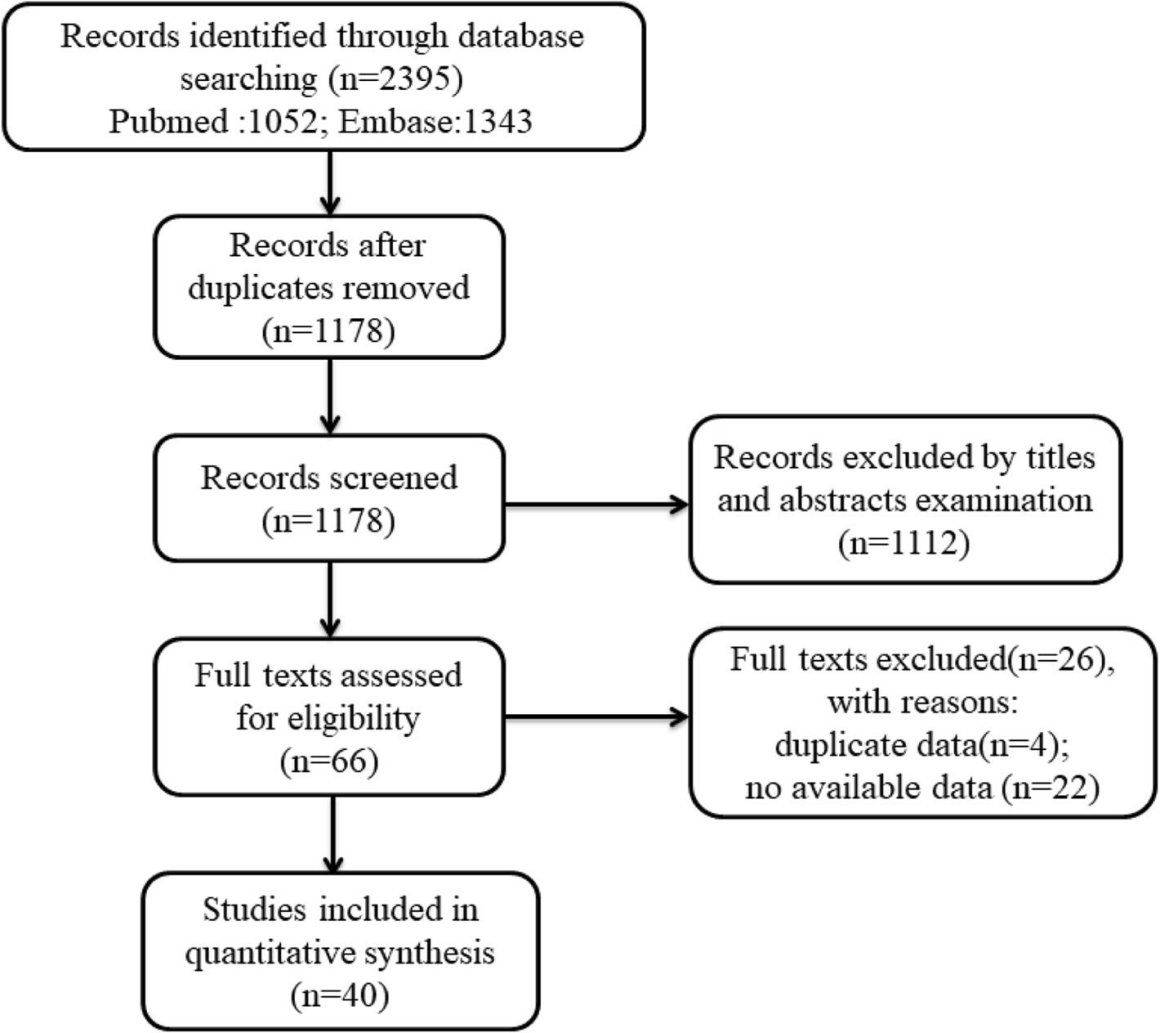
Flow chart representing the search steps and study selection

A flow diagram demonstrating the search procedure is illustrated in Fig. 1. After the original search, 2395 records were retrieved from the electronic databases. First, we removed duplications, and 1178 records remained. Among them, another 1112 records were also excluded after examining the titles and abstracts. Next, the remaining 66 full texts were assessed for eligibility. Of these, 26 studies were excluded on account of duplicate dates and incomplete data. Ultimately, 40 studies including a total of 16,696 participants met the criteria and were enrolled in this meta-analysis [17–19, 26–60].

The characteristics of the patients included are presented in Table 1. All included studies were published between 1976 and 2017. As shown in Table 1, 40 studies were included in the meta-analysis, 30 studies on NSCLC, 2 studies on SCLC, and 8 studies on all tumour types. Twenty-three studies were performed in Asian populations and 16 in Caucasian populations, while one study did not report the race of the participants. In terms of the cut-off value of platelet count, 8 studies used < 300 as the cut-off value, 18 studies used 300–400 as the cut-off value, one study did not report the cut-off value of platelet count, and the remaining 13 studies considered ≥400 as the cut-off value. There were 39 studies evaluating the association between OS and platelet count, while 13 studies evaluated the DFS/PFS/TTP outcome. All 40 studies reported the HR and 95% CI directly. Additionally, the quality of the studies was assessed by NOS, as shown in Table 2.

**Table 1:** The basic characteristics of included studies in the meta-analysis

Author	Year	Country	Cases	Tumor type	Clinical stage	Cut-off value	Outcome	OS		DFS/PFS/TTP	
								U/M	HR(95%CI)	U/M	HR(95%CI)
Pedersen LM	1996	Denmark	1115	NSCLC+ SLCL	I-IV	400	OS	M	4.24(1.50–12.72)		
Cox G	2000	UK	175	NSCLC	I-IIIA	320	OS	M	1.69(1.12–2.56)		
Suzuki M	2002	Japan	99	NSCLC	I-IV	231	OS	M	3.04(1.08–8.55)		
Swinson DE	2003	United Kingdom	175	NSCLC	I-IIIA	314	OS	M	1.64(1.13–2.39)		
Bremnes RM	2003	Norway	436	SCLC	–	150	OS	M	3.10(1.40–7.20)		
Unsal E	2004	Turkey	58	NSCLC+ SLCL	I-IV	400	OS	M	0.96(0.54–1.72)		
Aoe K	2004	Japan	611	NSCLC+ SLCL	I-IV	400	OS	M	1.29(1.02–1.64)		
Prévost S	2006	Canada	120	NSCLC	Not Report	340	OS	M	1.50(1.0–2.30)		
Tomita M	2008	Japan	240	NSCLC	I-IV	400	OS	M	1.46(1.01–2.01)		
Gonzalez Barcala FJ	2010	Spain	365	NSCLC+ SCLC	I-IV	258	OS	M	1.15(0.90–1.47)		
Gonzalez Barcala FJ	2010	Spain	294	NSCLC+ SCLC	I-IV	381	OS	M	1.09(0.82–1.46)		
Luo J	2012	USA	110	NSCLC	I-IV	300	OS	M	2.86(1.48–5.54)		
Holgersson G	2012	Sweden	823	NSCLC	I-IV	350	OS	M	1.35(1.12–1.62)		
Yu D	2013	China	510	NSCLC	I-III	300	OS,DFS	M	1.69(1.01–2.38)	M	1.57(1.01–2.45)
Maráz A	2013	Hungary	398	NSCLC+ SLCL	I-IV	400	OS	M	1.58(1.14–2.18)		
Kim KH	2014	Korea	854	NSCLC	III-IV	450	OS	M	1.51(1.14–2.00)		
Kim M	2014	Korea	199	NSCLC	I-III	400	OS,DFS	M	2.98(1.39–6.37)	M	2.47(1.22–5.01)
Ji Y	2014	China	234	NSCLC	I	300	OS,DFS	M	3.14(1.23–8.03)	M	5.31(2.75–10.27)
Zhu JF	2014	China	275	NSCLC	IV	300	OS	M	1.40(1.00–2.00)		
Hong X	2016	China	999	SCLC	–	300	OS,PFS	M	1.01(0.87–1.18)	M	0.91(0.70–1.17)
Gotfrit J	2016	Canada	223	NSCLC	IIIB-IV	400	OS	M	1.46(1.03–2.09)		
Boddu P	2016	USA	571	NSCLC	I-IV	450	OS	M	1.64(1.05–2.55)		
Liu W	2017	China	1120	NSCLC	I-IIIA	300	OS,DFS	M	1.15(0.96–1.39)	M	1.17(0.97–1.40)
Wang YQ	2017	China	134	NSCLC	I-IIIA	289	OS,DFS	M	2.28(1.43–3.62)	U	1.63(1.01–2.64)
Holgersson G	2017	Sweden	222	NSCLC	III	350	OS	M	1.66(1.12–2.48)		
Holgersson G	2017	Sweden	99	NSCLC	IIIB-IV	350	OS	M	1.25(0.71–2.22)		
Cui MM	2017	China	270	NSCLC	I-III	Not Report	OS	M	1.00(1.00–1.01)		
Ohuchi M	2017	Japan	146	NSCLC+ SLCL	I-IV	244	OS	M	1.88(1.13–3.13)		
Mandrekar SJ	2006	Canada+ USA	1053	NSCLC	IIIB-IV	375	OS,TTP	U	1.41(1.24–1.60)	U	1.27(1.11–1.45)
Altiay G	2007	Turkey	78	NSCLC+ SLCL	III-IV	400	OS	U	2.33(1.27–4.26)		
Qiu MZ	2010	China	430	NSCLC	I-IV	400	OS	U	1.09(0.60–1.98)		
Liu HB	2013	China	883	NSCLC	I-IV	300	OS	U	1.30(1.02–1.66)		
Du G	2013	China	258	NSCLC	IIIA-IV	400	OS,PFS	U	4.15(3.09–5.59)	U	3.47(2.60–4.65)
Zhang T	2014	China	400	NSCLC	I-II	190	OS,DFS	U	1.47(0.88–2.45)	U	1.57(1.01–2.45)
Wu G	2015	China	366	NSCLC	III-IV	117.5	OS,PFS	U	1.22(0.90–1.65)	U	1.25(0.92–1.69)
Zhang H	2015	China	1238	NSCLC	I-IIIA	300	OS,DFS	U	1.38(1.17–1.63)	U	1.38(1.16–1.63)
Zhang W	2015	China	308	NSCLC	I-IV	300	OS	U	1.67(1.23–2.27)		
Gao L	2017	China	546	NSCLC	I-IIIA	300	OS,DFS	U	1.72(1.35–2.19)	U	1.70(1.33–2.17)
Li Y	2014	China	126	NSCLC	III-IV	200	PFS			M	1.69(1.16–2.46)
Lee S	2017	Korea	135	NSCLC	IIIB-IV	400	OS	U	1.49(0.80–2.78)		

*NSCLC* Non-small cell lung cancer, *SCLC* Small cell lung cancer, *OS* Overall survival, *DFS* Disease-free survival, *PFS* Progress-free survival, *TTP* Time to progress, *HR* Hazard ratio, *CI* Confidence interval *M* Multivariate analysis, *U* Univariate analysis

**Table 2:** Quality assessment of containing studies using the Newcastle-Ottawa Scale

Study	Selection	Comparability	Outcome	Total score
Pedersen LM	4	1	3	8
Cox G	4	2	2	8
Suzuki M	4	0	2	6
Swinson DE	4	0	2	6
Bremnes RM	4	0	2	6
Unsal E	4	1	2	7
Aoe K	4	0	2	6
Mandrekar SJ	4	1	2	7
Prévost S	4	0	2	6
Altiay G	4	0	2	6
Tomita M	4	2	2	8
Qiu MZ	4	2	2	6
Gonzalez Barcala FJ	4	2	2	8
Gonzalez Barcala FJ	4	2	2	8
Luo J	4	1	2	7
Holgersson G	4	1	2	7
Yu D	4	2	2	8
Liu HB	4	0	2	6
Maráz A	4	0	2	6
Du G	4	1	2	7
Kim KH	4	2	1	7
Kim M	4	2	2	8
Zhang T	4	0	3	7
Ji Y	4	1	2	7
Zhu JF	4	2	2	8
Wu G	4	0	2	6
Zhang H	4	1	2	7
Zhang W	4	0	3	7
Hong X	4	0	2	6
Gotfrit J	4	0	2	6
Boddu P	4	1	2	7
Gao L	4	2	3	9
Liu W	4	2	2	8
Wang YQ	4	1	2	7
Lee S	4	0	2	6
Holgersson G	4	0	2	6
Holgersson G	4	0	2	6
Cui MM	4	0	3	7
Ohuchi M	4	0	2	6
Li Y	4	2	1	7

### Meta-analysis

#### OS

**Fig. 2:**
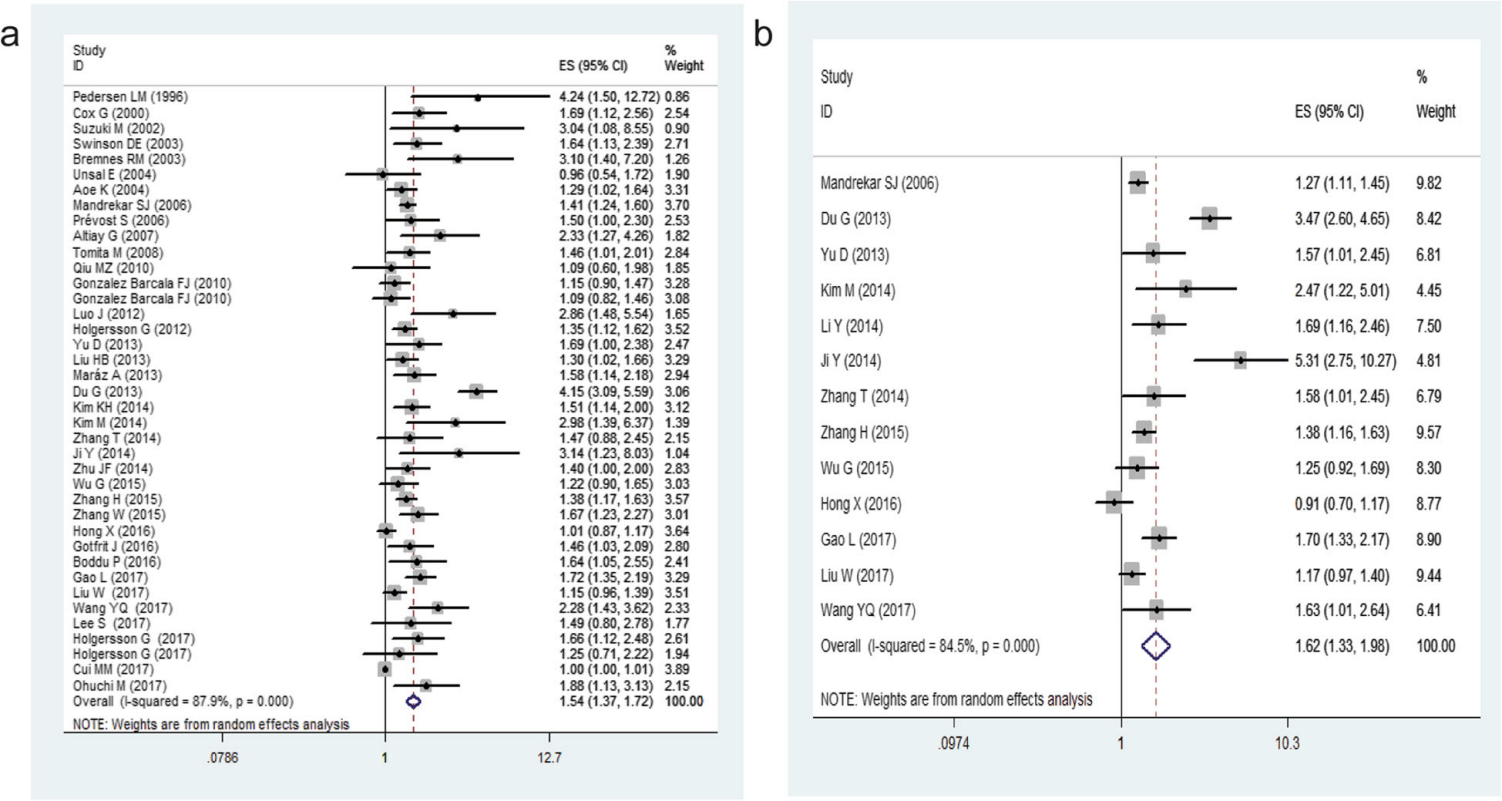
Forest plot showing the HRs with 95% CIs for the association between elevated platelet counts and OS (**a**) or DFS/PFS/TTP (**b**)

There were 39 studies including 16,570 patients providing data on the prognostic role of platelet count for OS in lung cancer. The results indicate that elevated platelet counts were associated with poorer OS in lung cancer patients (HR = 1.54, 95% CI: 1.37–1.72, *P* < 0.001, Fig. 2a). Then, we conducted subgroup analysis for further investigation, and the results are summarized in Table 3. In the subgroup stratified by ethnicity, we observed that elevated platelet counts predicted poor OS in Asian populations (HR = 1.54, 95% CI: 1.32–1.8, *P* < 0.001), while that in non-Asian populations had no significance (*P* = 0.063). Based on clinical stage, a significant association between elevated platelet counts and OS was found in stage I-III (HR = 1.52, 95% CI: 1.22–1.89, *P* < 0.001) and stage >III (HR = 1.7, 95% CI: 1.26–2.29, *P* < 0.001). An obvious association between elevated platelet counts and OS was observed when integrating the data from 28 studies in which OS was evaluated with multivariate analyses (HR = 1.47, 95% CI: 1.31–1.66, *P* < 0.001). In terms of the cut-off value, the subgroup analysis confirmed that increased platelet counts were a negative predictor in patients with cut-off values< 300 (HR = 1.64, 95% CI: 1.25–2.15, *P* < 0.05) and with cut-off values> 400 (HR = 1.73, 95% CI: 1.35–2.21, *P* < 0.001) Additionally, high platelet counts still predicted worse OS in patients with lung cancer, regardless of the subtype of lung cancer (SCLC or NSCLC).

**Table 3:** The results of subgroup analysis in meta-analysis of OS and DFS/PFS/TTP

	Subgroup	No. of studies	HR (95% CI)	P	Heterogeneity		Model used
					I^2^	P_h_	
OS	Analysis of variable						
	Multivariate	28	1.47(1.31–1.66)	< 0.001	80.60%	< 0.001	Random
	Univariate	11	1.62(1.33–1.99)	< 0.001	81.70%	< 0.001	Random
	Ethnicity						
	Asian	22	1.54(1.32–1.80)	< 0.001	89.80%	< 0.001	Random
	non-Asian	17	1.42(1.32–1.53)	0.063	37.00%	0.063	Fixed
	Tumor stage						
	I-III	9	1.52(1.22–1.89)	< 0.001	89.00%	< 0.001	Random
	III-IV	8	1.70(1.26–2.29)	< 0.001	85.80%	< 0.001	Random
	I-IV	15	1.37(1.26–1.49)	0.066	38.20%	0.066	Fixed
	Histology						
	NSCLC	29	1.58(1.38–1.82)	< 0.001	89.80%	< 0.001	Random
	SCLC	2	1.64(0.55–4.87)	0.008	85.60%	0.008	Random
	NSCLC+SCLC	8	1.39(1.14–1.70)	0.036	53.40%	0.036	Random
	cut-off value						
	<300 × 10^9/L	7	1.64(1.25–2.15)	0.024	58.80%	0.024	Random
	300 × 10^9/L ≤ cut-off value<400 × 10^9/L	18	1.40(1.27–1.55)	0.002	55.40%	0.002	Random
	≥400 × 10^9/L	13	1.73(1.35–2.21)	< 0.001	77.90%	< 0.001	Random
	Quality score						
	> 6	23	1.59(1.36–1.85)	< 0.001	91.20%	< 0.001	Random
	≤6	16	1.30(1.19–1.41)	0.015	48.60%	0.015	Fixed
DFS/PFS/TTP	Analysis of variable						
	Multivariate	7	1.66(1.14–2.42)	< 0.001	84.40%	< 0.001	Random
	Univariate	6	1.63(1.28–2.09)	< 0.001	85.50%	< 0.001	Random
	Ethnicity						
	Asian	12	1.68(1.33–2.12)	< 0.001	85.10%	< 0.001	Random
	non-Asian	1	–	–	–	–	–
	Tumor stage						
	I-III	6	1.40(1.26–1.55)	0.097	46.30%	0.097	Fixed
	III-IV	4	1.74(1.09–2.78)	< 0.001	92.50%	< 0.001	Random
	Histology						
	NSCLC	12	1.71(1.40–2.09)	< 0.001	83.00%	< 0.001	Random
	SCLC	1	–	–	–	–	–
	cut-off value						
	<300 × 10^9/L	4	1.47(1.21–1.78)	0.584	0.00%	0.584	Fixed
	300 × 10^9/L ≤ cut-off value<400 × 10^9/L	7	1.42(1.15–1.74)	< 0.001	81.40%	< 0.001	Random
	≥400 × 10^9/L	2	3.30(2.52–4.32)	0.383	0.00%	0.383	Fixed
	Quality score						
	> 6	11	1.77(1.42–2.20)	< 0.001	84.30%	< 0.001	Random
	≤6	2	1.04(0.85–1.26)	0.114	0.599	0.114	Fixed

*OS* Overall survival, *DFS* Disease-free survival, *PFS* Progress-free survival, *TTP* Time to progress, *NSCLC* Non-small cell lung cancer, *SCLC* Small cell lung cancer, *HR* Hazard ratio, *CI* Confidence interval

#### DFS/PFS/TTP

The meta-analysis of DFS/PFS/TTP, which contained 13 studies with 7183 patients, indicated that cancer patients with high platelet counts had significantly shorter DFS/PFS/TTP than those with low platelet counts (HR = 1.62, 95% CI: 1.33–1.98, *P* < 0.001, Fig. 2b). A random-effects model was used. Subgroup analysis was performed, and the results are shown in Table 3. The results suggested that in the subgroup analysis, elevated platelet count was a negative predictor in the Asian population subgroup (*P* < 0.001), multivariate analysis subgroup (*P* < 0.001), stage III-IV disease subgroup (*P* < 0.001), and 300 ≤ cut-off value< 400 subgroup (*P* < 0.001).

In the following four subgroups, patients with elevated pretreatment platelet counts had similar DFS/PFS/TTP compared with patients with normal platelet counts: stage I-III disease subgroup (*P* = 0.097), studies with a quality score ≤ 6 (*P* = 0.114), platelet count > 400 subgroup (*P* = 0.383), and platelet count < 300 subgroup (*P* = 0.584).

#### Publication bias and sensitivity analysis

**Fig. 3:**
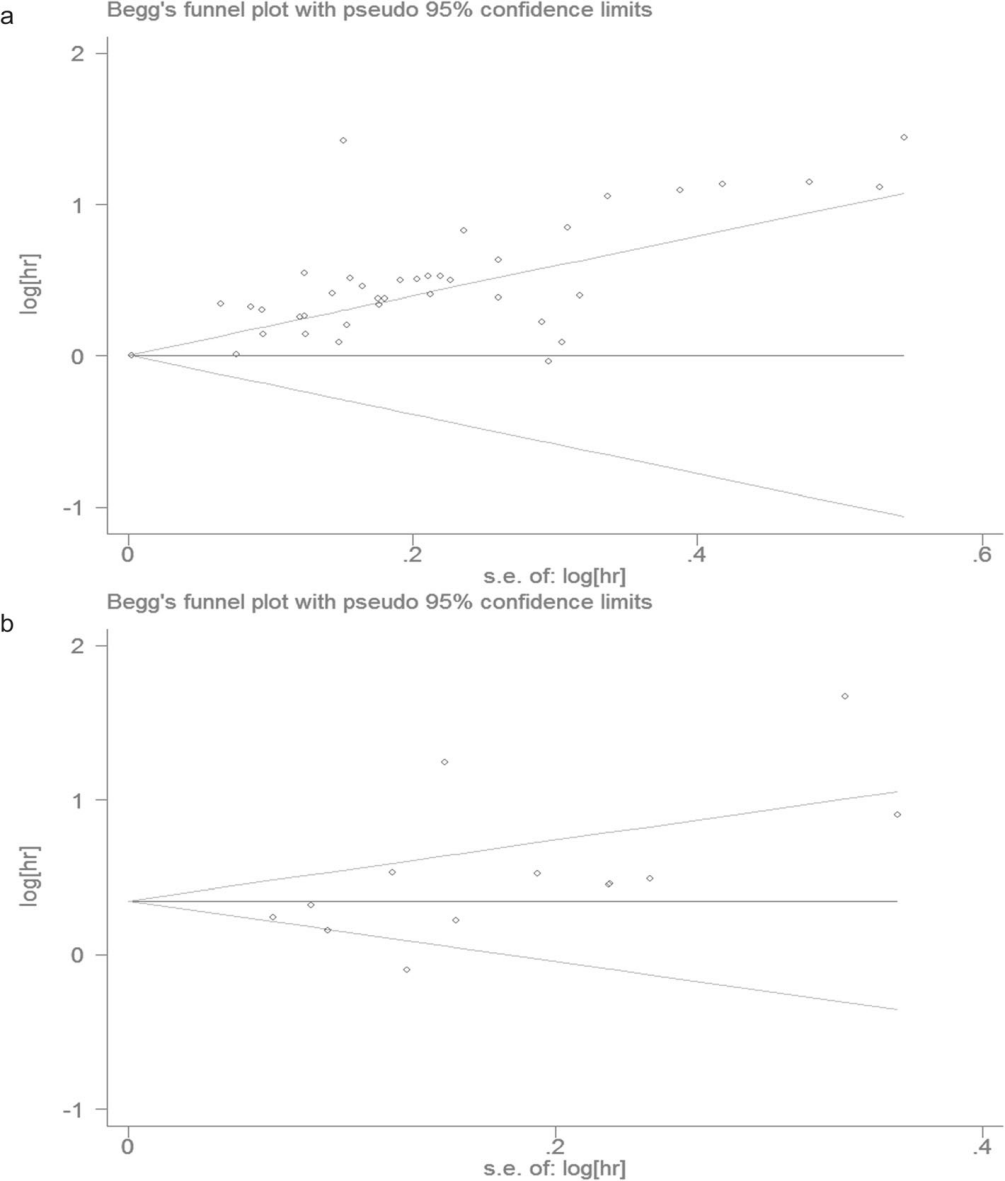
Begg’s funnel plots and Egger’s test evaluating possible publication bias for (**a**) OS; (**b**) DFS/PFS/TTP

As shown in Fig. 3, the funnel plot was symmetrical. Based on Begg’s test (*P* = 0.866) and Egger’s regression test (*P* = 0.376), no significant publication bias was found.

**Fig. 4:**
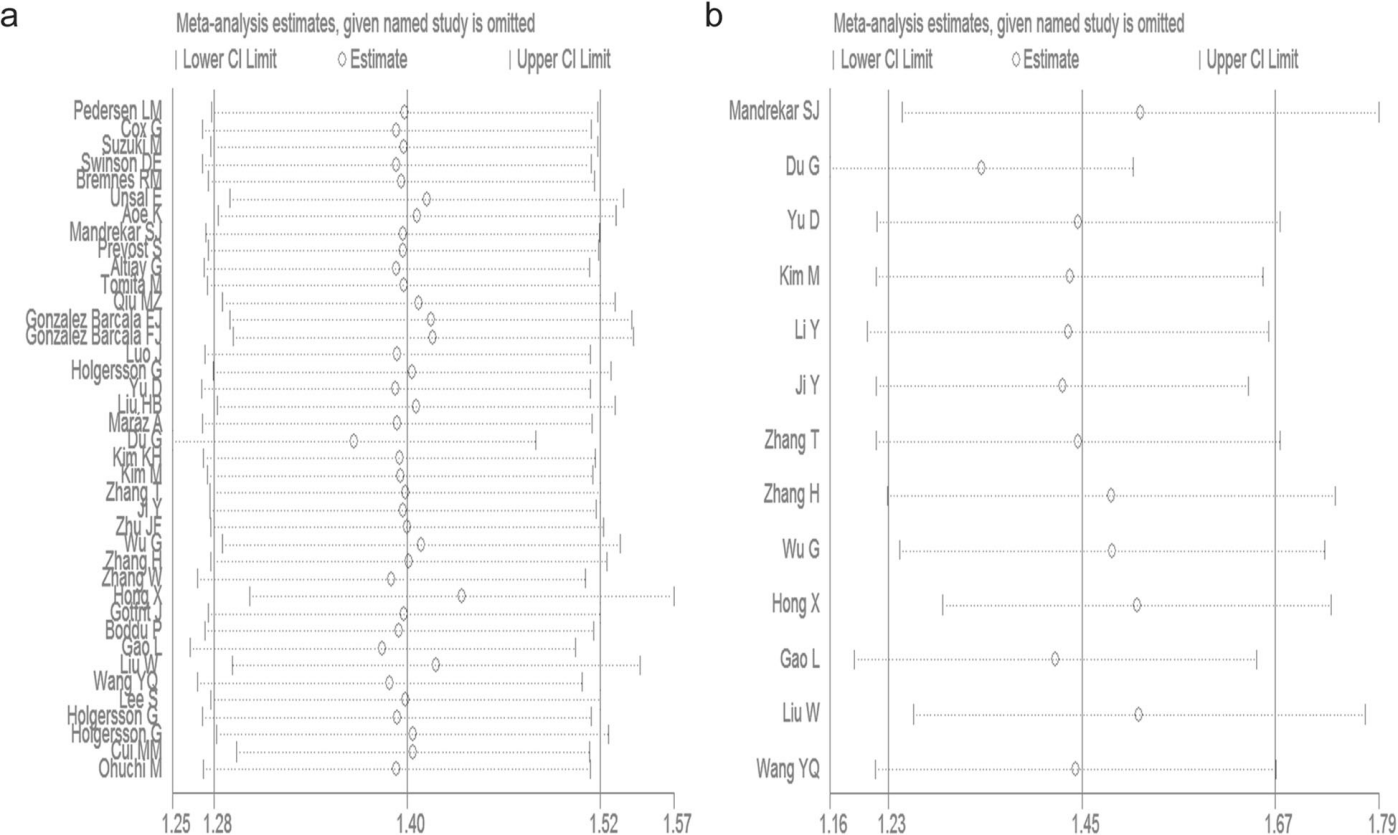
Sensitivity to the relationship between elevated platelet counts and OS (**a**) or DFS/PFS/TTP (**b**)

Furthermore, we performed sensitivity analysis to evaluate the reliability of our results. The corresponding pooled HR values were not significantly impacted, indicating the robustness of our conclusions (Fig. 4).

## Discussion

Cancer is undoubtedly one of the most serious public health problems. In the past few years, antidiuretic hormone (ADH), tumour necrosis factor alpha (TNF-α), NF-κB/p65, COX-2 and thyroid transcription factor-1 (TTF-1) have been reported to be associated with the prognosis of lung cancer. However, their specificity and sensitivity in prognosis are still not satisfactory. Therefore, the exploration of new lung cancer prognostic markers is of great significance for clinicians to take targeted measures and improve the prognosis of patients.

In recent years, it has been observed that some systemic inflammation indicators, such as the neutrophil-to-lymphocyte ratio (NLR) [61], platelet-to-lymphocyte ratio (PLR) [62], Glasgow prognostic score (GPS) [63], Prognostic Index (PI) and Prognostic Nutritional Index (PNI) [64], play important roles in tumorigenesis and development and can be considered predictors of prognosis. In the 1960s, Richard B observed that the platelet count is elevated in patients with cancers compared to those with nonmalignant diseases [5]. Accumulating evidence suggests that elevated platelet counts are associated with various cancers, such as colorectal cancer, lung cancer, and endometrial carcinoma [12, 65, 66]. Platelets sustain proliferative signalling, resist cell death, and induce tumour angiogenesis [67]. Additionally, platelets activate the TGF-β/Smad and NF-κB pathways, further promoting tumour migration and invasion [68]. Moreover, as immune cells [69], platelets release TGF-β, reducing the expression of NKG2D and weakening the role of natural killer (NK) cells [70]. Platelets could be a prognostic predictor used in the clinic. Recently, several studies confirming the prognostic value of platelet count in lung cancer have been carried out; however, the results were inconsistent. Therefore, we conducted a meta-analysis to determine the precise role of platelet counts in lung cancer.

We combined the outcomes of 40 studies with 16,696 patients, suggesting that elevated platelet counts are a poor predictor of OS and DFS/PFS/TTP in lung cancer patients. In our subgroup analysis, elevated platelet counts were significantly associated with poor OS and DFS/PFS/TTP in diverse subgroups, such as Asian populations, tumour stages I-III, tumour stages III-IV, and studies with quality scores > 6. However, the cut-off value of the platelet count was variable. We found that elevated platelet counts were significantly associated with poor OS and DFS/PFS/TTP when the cut-off value was between 300 and 400, while the cut-off value of > 400 did not have a relationship with poor DFS/PFS/TTP. Overall, the cut-off value of plate count between 300 and 400 can separate patients well for OS and DFS/PFS/TTP and should be used as a prognostic biomarker in clinical use, which is more precise than the findings of the previous meta-analysis [71]. Compared to the previous meta-analysis [71], our results are more comprehensive and accurate. On the one hand, we included 40 articles in the meta-analysis, which included more new and important studies, increasing the analytical capability of the analysis. On the other hand, a more detailed subgroup analysis was performed. In addition to race and the cut-off value of platelet count, we also investigated the prognostic role of platelet count in different tumour stages, histology and quality scores. Additionally, we discussed the association between platelets and OS as well as DFS/PFS/TTP, while the previous meta-analysis studied only the significance in OS.

However, there are some limitations of this study that deserve to be mentioned. First, the studies included in our meta-analysis are retrospective studies and are therefore more likely to have selection bias. Second, although the publication bias and sensitivity analyses confirmed the credibility of our analysis, heterogeneity still existed in this meta-analysis due to several factors, such as patient characteristics, sample size, and adjuvant therapy, which were not included in our analysis. Moreover, the cut-off value for definition of the elevated platelet counts differed among the studies. Most of the studies used 300–400 as the cut-off value, while several others used < 300 or > 400 as the cut-off value of platelet count to assess the prognosis, which might lead to between-study heterogeneity. Last, platelet count could be affected by several factors, such as thrombosis, hypertension, splenic diseases, blood coagulation disorders, myeloproliferative disease, infection and drugs. Therefore, platelet count cannot play a prognostic role if patients have these diseases mentioned above.

## Conclusions

In conclusion, our meta-analysis revealed that elevated pretreatment platelet counts are related to poor OS and DFS/PFS/TTP in lung cancer patients and are an independent prognostic predictor of lung cancer patients. Considering the limitations, large-scale prospective studies and a validation study are warranted to confirm our results.

## Supplementary information

**Additional file 1.** PRISMA checklist.

## Data Availability

The data used and/or analysed during the current study available from the corresponding author on reasonable request.

## Abbreviations

DFS: Disease-free survival
PFS: Progression-free survival
TTP: Time to progression
OS: Overall survival
HR: Hazard ratio
CIs: Confidence intervals
NSCLC: Non-small-cell lung cancer
SCLC: Small cell lung cancer
NOS: Newcastle-Ottawa Scale
NLR: Neutrophil-to-lymphocyte ratio
PLR: Platelet-to-lymphocyte ratio
GPS: Glasgow prognostic score
PI: Prognostic Index
PNI: Prognostic Nutritional Index
